# 

**Published:** 1808-08

**Authors:** 


### NEW MEDICAL PUBLICATIONS.

The Syphilitic Physician; being a Treatise on the Venereal Disease. By C. Erskine, Surgeon. 2s.

The Chirurgical Candidate; or Reflections on Education, indispensable to complete Naval, Military, and other Surgeons. By Charles Dunne, Member of the Royal College of Surgeons. 8vo. 10s. 6d.

The Chirurgical Works of the late Percival Pott, F. R. S. A new edition, containing his last corrections; with Notes, and a short Account of the Life of the Author. By Sir James Earle, F. R. S. Surgeon Extraordinary to the King. 3 vol. 8vo. 1l. 7s.

The Medical Remembrancer, or Pharmaceutical Vade Mecum. By Thomas Churchill, Apothecary. 2s.

### To CORRESPONDENTS.

If "Honestas" will take the trouble to call at No. 21, Greville Street, Hatton Garden, Mr. Taunton will answer his question.

